# Lifestyle Risk Factors and the Population Attributable Fractions for Overweight and Obesity in Chinese Students of Zhejiang Province

**DOI:** 10.3389/fped.2021.734013

**Published:** 2021-09-22

**Authors:** Fang Gu, Siliang Zhou, Ke Lou, Rui Deng, Xingxiu Li, Jie Hu, Bin Dong

**Affiliations:** ^1^Institute for Nutrition and Health, Zhejiang Provincial Center for Disease Control and Prevention, Hangzhou, China; ^2^Institute of Child and Adolescent Health, School of Public Health, Peking University Health Science Center, Beijing, China; ^3^Menzies Health Institute Queensland, Griffith University, Brisbane, QLD, Australia

**Keywords:** obesity, student, lifestyle factors, population attributable fraction, Zhejiang province

## Abstract

**Objectives:** To assess the relationship between modifiable lifestyle factors and risk of overweight/obesity in Chinese students, and to evaluate the predicting prevalence of overweight if the lifestyle risk factors were removed.

**Methods:** A cross-sectional survey was conducted among 40,141 students in grade three and above (8–24yrs) in 2019 in Zhejiang Province, China. Physical examination was performed, and a self-administered questionnaire was used to collect lifestyle information, including dietary behavior, physical activity, TV watching, sleeping, smoking, drinking, and tooth-brushing habits. Logistic regression models were performed to assess the relationship between overweight/obesity and a series of lifestyle factors. Population attributable fractions (PAFs) were used to calculate the predicting prevalence of overweight/obesity if lifestyle risk factors were removed.

**Results:** The prevalence of overweight/obesity of participants was 25.5% (male 32.3%, female 18.1%). Overweight/obesity were associated with adverse lifestyle factors, such as watch TV ≥1 h/day (OR = 1.14, 95% CI: 1.11–1.22), insufficient sleep (OR = 1.14, 95% CI: 1.11–1.22), and irregular toothbrushing habits (OR = 1.19, 95% CI: 1.01–1.39). Based on the calculated PAFs, the predicted prevalence of overweight/obesity would decline moderately if lifestyle factors were modified, with the magnitudes of decrease vary by sex, age and residence. Generally, a larger reduction was estimated if the sleeping time was increased and TV time was reduced, with the prevalence of overweight/obesity decreased by 1.1% (95% CI: 0.7, 1.5%) and 0.9% (95% CI: 0.6, 1.2%), respectively.

**Conclusions:** Predicted prevalence of overweight/ obesity in Chinese students may decrease if modifiable lifestyle risk factors were removed. The attributable risk for obesity of lifestyle behaviors varied in age, sex and residence groups. The findings of this study may provide insights for planning and optimizing future obesity intervention endeavors.

## Introduction

The worldwide prevalence of childhood obesity has increased dramatically during the past decades (1). Among children and adolescents aged 5–19, the prevalence of overweight and obesity rose from 4% in 1975 to over 18% in 2016 (2). Overweight/obesity in childhood is associated with adverse short-term and long-term health consequences, including hypertension (3), type two diabetes (4), musculoskeletal disease (5), and premature death (6, 7). Studies suggested that around three-quarters of children with overweight or obesity could track into adulthood (8). Intervention for obesity at the early stage of life has the potential to reduce the risk of adult obesity and obesity-related diseases in the future (9).

Understanding lifestyle factors for obesity intervention in children and adolescences are necessary. The attribution of these factors, such as dietary behavior, physical activity, screen time and sleeping, could vary in different populations (10–13). For examples, the consumption of sugar-sweetened beverage has a greater impact on obesity in male Korea adults than in their female counterparts (10). Inadequate sleeping has a greater impact on obesity in 9–13 years old boys than in 4–8 years old boys (14). The cumulative population attributable fraction (PAF) for childhood obesity was 42.9% in a cohort study in New Zealand, as if the excessive screen time and several other related factors were eliminated (15). Furthermore, a study found that not participating in sports' teams (PAF, 16.57%; 95% CI, 15.30–17.84%) had the biggest influence on the rate of obesity in American high school students, followed by watching TV and playing computer games (16).

Overall, modifiable lifestyle factors include but not limited to the aforementioned factors, were identified to be significantly associated with obesity (10, 13, 17, 18). However, few intervention programs pragmatically endeavored to reduce multiple risk behaviors. A sense of urgency to address the obesity epidemic has contributed to the demands for immediate advocacy based upon the best available evidence (19). This study aimed to identify the lifestyle risk factors and to quantify the attribution of the preventable lifestyle factors to overweight and obesity in Chinese children and adolescents. The findings of this study may be valuable for prioritizing the intervention effort to reducing the risk of obesity.

## Methods

### Study Population

This study used data collected in a school-based health survey in Zhejiang Province in 2019. Students of Grade three and above (8–24 yrs) were recruited from 11 cities of the province. Random cluster sampling was conducted in this survey. One urban area and one rural area were selected from each city, with 8 schools (two primary schools, 2 middle schools, 2 high schools, 1 vocational high school, and 1 university) and five schools (two primary schools, two middle schools, and one high school) randomly selected from the urban area and the rural area, respectively. School classes from third grade and over were enrolled until the number of participants was no <80 for each grade. There is no age, sex, height, weight or residence (urban/rural residing) missing in our data set. Furthermore, all participants' heights and body weights did not exceed five times the standard deviation of the age-and sex-specific means in our data set. The sample size of the study is 40,141.

### Measures

All participants underwent the physical examination according to a standard survey protocol. Weight and height were measured when participants had removed shoes and heavy clothes. Height (measured to the nearest 0.1 cm) and weight (measured to the nearest 0.1 kg) were measured using standardized equipment. All measurements and physical examinations were conducted separately for males and females. The BMI was calculated by dividing the weight in kilogram with the height square expressed in meters (kg/m^2^).

In children and adolescents aged between 8 and 18 years, WHO standards were used and participants were classified as normal weight (BMI Z score <1) and overweight/obesity (BMI Z score >1) (20). Participants over 18 years old were categorized as normal weight for BMI <25 kg/m^2^ and overweight/obesity for BMI ≥ 25 kg/m^2^, respectively.

The questionnaire which was developed and validated for the annual national project “Surveillance for common disease and health risk factors among students” since 2016, was used to collect lifestyle behaviors information in the present study. The validity of the questionnaire has been published previously (21, 22). Three sets of questionnaires were used in students at primary schools, middle/high schools, and universities. Similar questions were included in these questionnaires with different wording that was appropriate to the age group. Questionnaires were completed by students themselves with the help of class teachers if required.

The lifestyle factors, including sugar-sweetened beverages consumption (<1 time/day and ≥1 time/day), intake if fried food (0 time/day or ≥1 time/day), breakfast (0 time/day or ≥1 time/day), toothbrushing (<1 time/day or ≥1 time/day), physical activity (<1 time/week or ≥1 time/week), watching TV (0–1 h daily or ≥1 h daily) were categorized into two groups. These classifications were based on previous studies conducted among the Chinese population (17), as well as the data distribution of lifestyle factors. Sleep duration was dichotomized as adequate or inadequate, according to the recommendation of the National Sleep Foundation (23). For 9–13 years, at least 9 h of sleep per day is considered adequate, and <9 h of sleep per day is considered inadequate. The corresponding cut-off values for adolescents of 14–17 years and young adults of 18–25 years were 8 and 7 h, respectively. Participants were also asked whether smoked during the past 30 days, as well as whether ever had one glass of wine.

### Statistical Analysis

Descriptive statistics were calculated for all variables. Logistics regression models were performed to assess the relationship between overweight/obesity and lifestyle factors when age, sex, and urban/rural area were adjusted for. The multicollinearity test found that there is no significant collinearity between the variables.

PAF is most often defined as the proportion of disease incidence, or of disease risk, that would be eliminated from the population if exposure to the risk factor was eliminated (24). It is often used for clarity or justification for causal apportioning, hypothesis about the effects of preventive interventions (25). The PAF (%) and its 95% confidence intervals (CI) were estimated based on asymptotic approximations from a logistic regression model, using the formula suggested by Greenland and Drescher (26). After adjusting for age, sex, city, and urban/rural area, the PAFs were presented by age, sex, and urban/rural area groups. The theoretical prevalence of overweight/obesity, if the risk factor was eliminated, was estimated using the actual prevalence of overweight/obesity multiplied by (1-PAF%). Data were analyzed using R version 4.0.3 (https://www.Rproject.org) and Stata SE 16 (Stata Corp LLC).

### Ethics Issues

Informed consent was obtained from all participants and their parents if participants were under 18 years old. The present study has been approved by Peking University Institutional Review Board (IRB00001052-20027-免).

## Result

A total of 20,801 (51.8%) males and 19,340 (48.2%) females participants aged 9–24 years were analyzed. As shown in Table 1, the prevalence of overweight/obesity is higher in males (32.3%) than in females (18.1%) and is slightly higher in participants living in the rural area (27.0%) compared to those living in the urban area (24.5%). Overall, compared with those of non-overweight, students with overweight/obesity had worse lifestyle behaviors, with higher rates of sugar-sweetened beverages consumption ≥1 time/day (*P* = 0.004), TV time ≥1 h/day (*P* < 0.001), never drunk (*P* = 0.001), toothbrushing habits <1 time/day (*P* < 0.001), and more physical activity (*P* < 0.001).

**Table 1:** Participants' demographic characteristics and lifestyle behaviors.

**Variables**	**Normal weight**	**Overweight/obesity**	**Total**	***P-*value**
Age, year^a^	14.2 (3.0)	13.3 (2.8)	14.0 (3.0)	<0.001
Height, cm	157.7 (12.6)	158.3 (12.3)	157.8 (12.6)	<0.001
Weight, kg	47.0 (11.9)	62.5 (16.5)	51.0 (14.8)	<0.001
Sex^b^				<0.001
Male	14,081 (67.7)	6,720 (32.3)	20,801	
Female	15,840 (81.9)	3,500 (18.1)	19,340	
Residence^b^				<0.001
Urban	18,562 (75.5)	6,022 (24.5)	24,584	
Rural	11,359 (73.0)	4,198 (27.0)	15,557	
Sugar-sweetened beverages^b^				0.004
≥1 time/day	2,922 (9.8)	1,101 (10.8)	4,023	
Fried food^b^				0.08
≥1 time/day	26,615 (89.0)	9,024 (88.4)	35,639	
Breakfast^b^			0.402
≥1 time/day	4,640 (15.5)	1,549 (15.2)	6,189	
Physical activity^b^				<0.001
< 1 times/week	5099 (17.1)	1,514 (14.8)	6,613	
Alcohol use^b^				0.001
YES	7748 (26.0)	2,476 (24.3)	10,224	
TV time^b^				<0.001
≥1 h/day	10,759 (36.0)	3,975 (39.0)	14,734	
Sleep duration^b^				0.246
Inadequate	13,810 (46.5)	4,778 (47.2)	18,588	
Toothbrushing habit^b^				<0.001
< 1 time/day	467 (1.6)	265 (2.6)	732	

a *Values are mean (sd)*.

b *Values are n (%)*.

As shown in this, sugar-sweetened beverages ≥1 time/day (OR = 1.14, 95% CI 1.06–1.23), toothbrushing habits < 1 time/day (1.07, 1.01–1.12), breakfast habits < 1 time/day (1.16, 1.08–1.24), alcohol use (1.12, 1.05–1.18), watching TV ≥ 1h/day (1.15, 1.10-1.21), insufficient sleep (1.16, 1.11–1.22) were associated with an elevated risk of overweight/obesity. These results were not significantly changed when age, sex, city, urban/rural area, and all other lifestyle factors were adjusted for.

**Figure 1:**
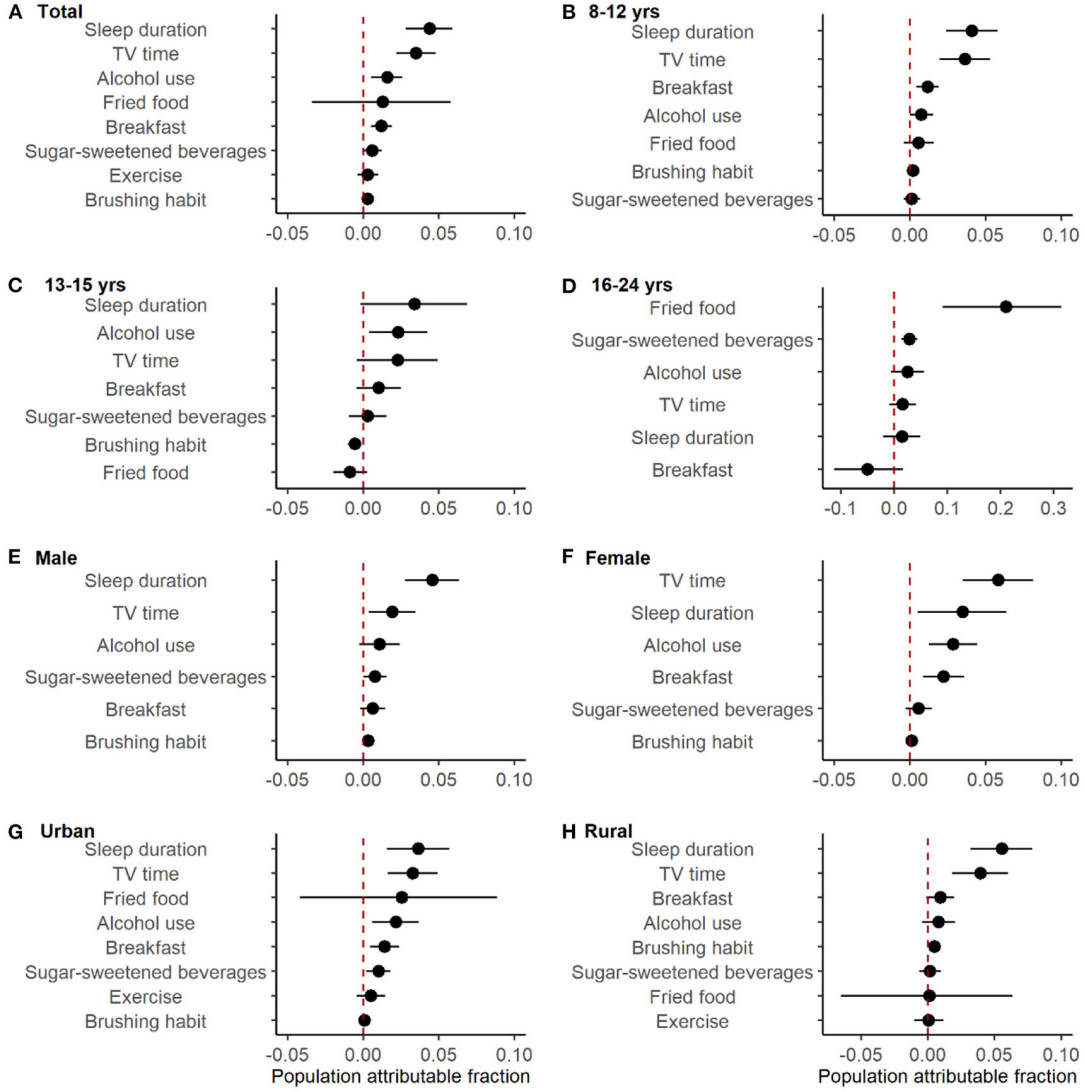
Adjusted population attributable fractions of lifestyle factors for overweight/obesity. Models were adjusted for age, sex, city, urban/rural area and all other factors. Dots were the estimated values. Error bars were 95% confidence intervals. **(B, C)** exercise wasn't shown because it was insignificant and had too wide CI to be shown. **(D)** toothbrushing habit wasn't shown because it was insignificant and the number of people who occasionally or didn't brush their teeth every day was small (54/11205). **(E, F)** fired food and exercise weren't shown because they were insignificant and had too wide CI to be shown.

Adjusted PAFs were calculated to quantify the contribution of each lifestyle factors to overweight/obesity (Figure 1 and Table 1). After controlling for age, sex, city, residence, and all other lifestyle factors, insufficient sleep time had the largest PAF (adjusted PAF: 4.4%, 95% CI: 2.8–5.9%), followed by prolonged TV time (adjusted PAF: 3.5%; 95% CI: 2.2–4.8%) and alcohol use (adjusted PAF: 1.6%; 95% CI: 0.5–2.6%).

Among different age, sex and residence groups, the magnitudes of PAFs varied. For example, consuming fried food showed the largest PAF in participants of 16–24 years old, with an adjusted PAF of 21.1% (95% CI, 9.1–31.4%). However, in those of 8–12 years old, sleep time was associated with the largest PAF (adjusted PAF: 4.1%; 95% CI: 2.4–5.8%). The risk factor with the largest PAF was prolonged TV time (adjusted PAF: 5.8%; 95% CI: 3.5–8.1%) in females and insufficient sleep in males (adjusted PAF:4.6%; 95% CI: 2.8–6.3%), respectively.

**Figure 2:**
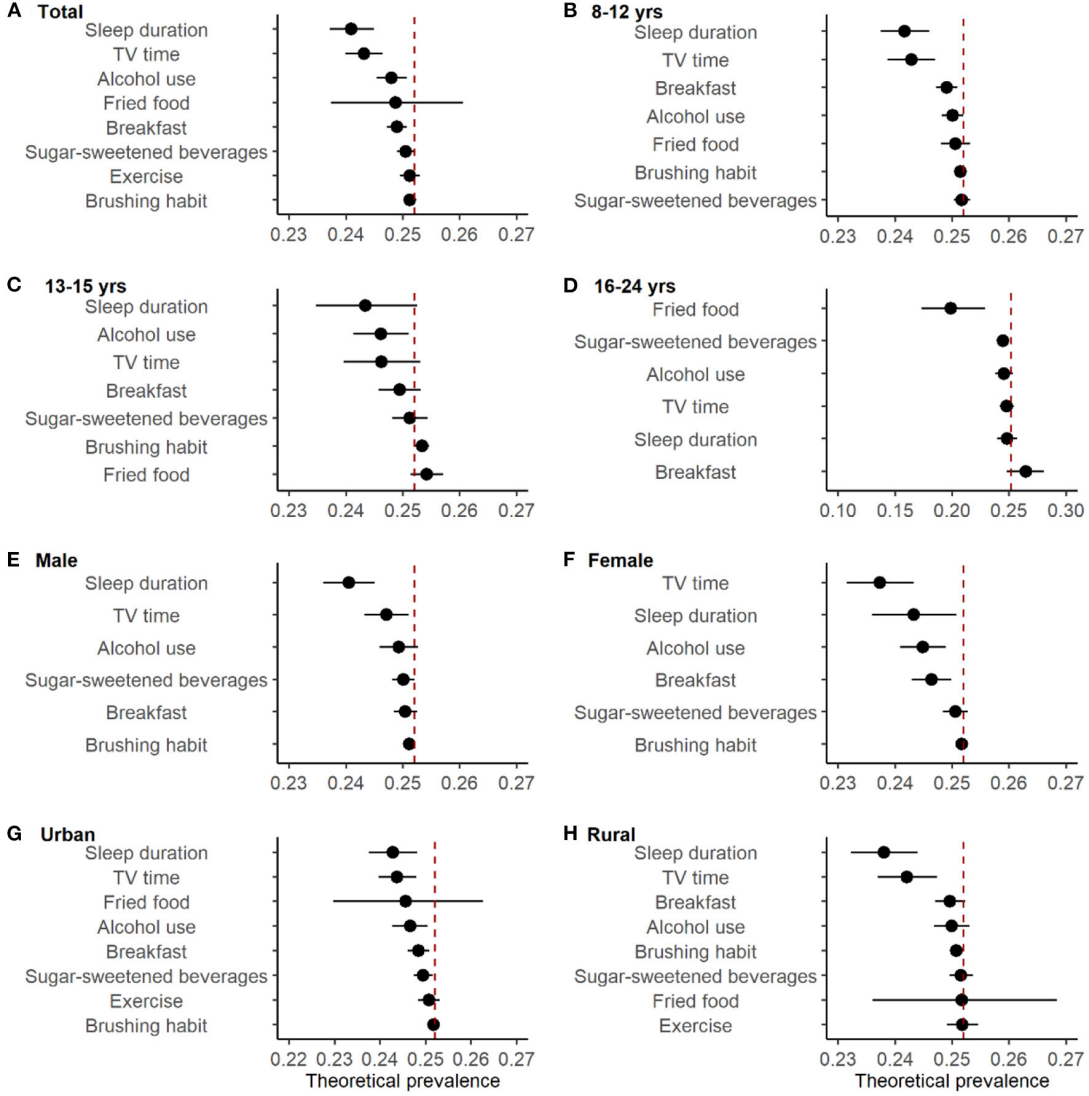
Predicting prevalence of overweight/obesity if the risk factor was removed, ranked by the magnitude of rate deduction. Models were adjusted for age, sex, city, urban/rural area and all other factors. Dots were the estimated values. Error bars were 95% confidence intervals. **(B, C)** exercise wasn't shown because it was insignificant and had too wide CI to be shown. **(D)** toothbrushing habit wasn't shown because it was insignificant and the number of people who occasionally or didn't brush their teeth every day was small (54/11205). **(E, F)** fired food and exercise weren't shown because they were insignificant and had too wide CI to be shown.

The predicting overweight/obesity prevalence, if each risk factor was eliminated, was additionally estimated (Figure 2 and Table 2). As shown in Figure 2, elimination of insufficient sleep duration, prolonged TV time, and alcohol use were associated with a larger reduction in overweight and obesity prevalence theoretically, with the prevalence drop by 1.1% (95% CI 0.7, 1.5%), 0.9% (95% CI 0.6, 1.2%), and 0.4% (95% CI 0.1, 0.7%), respectively. Among different age, sex and urban/rural area groups, the magnitude of theoretical reduction varied. For instance, in participants aged 13–15 years, alcohol use was associated with a larger reduction theoretically, with the prevalence declined by 0.6% (95% CI 0.1, 1.1%). However, in participants aged 13–15 years, fried food was associated with a larger reduction in overweight and obesity prevalence theoretically, and the corresponding decline was 5.3% (95% CI 2.3, 7.9%). For female participants, reduce TV time to <1 h was associated with the largest reduction of the predicting prevalence, which dropped by 1.5% (95% CI 0.8, 2.0%). While for male participants, ensuring adequate sleep time was associated with the largest reduction of the predicting prevalence, which dropped by 1.5% (95% CI 0.8, 2.0%).

**Table 2:** Associations between preventable lifestyle factors and overweight/obesity.

**Variables**	**Crude model**			**Adjusted model**		
	**OR**	**95% CI**	***P*-value**	**OR**	**95% CI**	***P*-value**
Sugar-sweetened beverages						
≥1 time/day (reference: <1 time/day)	1.12	1.04, 1.20	0.003	1.09	1.00, 1.17	0.039
Fried food						
≥1 time/day (reference: <1 time/day)	0.94	0.87, 1.01	0.077	1.02	0.95, 1.10	0.589
Breakfast						
1 time/day (reference: <1 time/day)	0.97	0.91, 1.04	0.393	1.12	1.05, 1.20	0.001
Physical activity						
< 1 times/week (reference: ≥1 time/week)	0.85	0.79, 0.90	<0.001	1.03	0.96, 1.10	0.441
Alcohol use						
YES (reference: NO)	0.92	0.87, 0.97	0.001	1.09	1.03, 1.16	0.003
TV time						
≥1 h/day (reference: < 1 h/day)	1.14	1.09, 1.19	<0.001	1.14	1.09, 1.20	<0.001
Sleep duration						
Inadequate (reference: adequate)	1.16	1.11, 1.22	<0.001	1.14	1.09, 1.20	<0.001
Toothbrushing habit						
< 1 time/day (reference: ≥ 1 time/day)	1.19	1.13, 1.25	<0.001	1.19	1.01, 1.39	0.037

*OR, odds ratio. Ref., reference. Adjusted Model was adjusted for age, sex, city, urban/rural area and all other factors in this table*.

## Discussion

This study quantified the contribution of preventable lifestyle factors for obesity intervention in Chinese students aged 9–24. Our study found that 37.3% of obesity could be prevented if insufficient sleep, prolonged TV time and alcohol use, which showed the top three largest PAFs, were eliminated. Based on the PAFs, if lifestyle factors were modified, the theoretical prevalence of overweight/obesity could decline moderately, though the magnitude of decline varied by sex, age and urban/rural area. Our study suggested that the estimation of PAFs could be useful to quantify the burden of overweight and obesity risk factors, which could potentially support the prioritization of national public health initiatives for obesity prevention in the future.

Overall, the prevalence of obesity/overweight was higher in males than that in females in this study, which was similar to what was found in other populations (27, 28). Our findings were also consistent with previous studies, which showed that unhealthy lifestyle behaviors, including poor oral hygiene (13), irregular breakfast habits (18), alcohol use (29), excessive TV time (12, 18, 30), and insufficient sleep time (11, 31) were associated with obesity in students (18). The mechanism why skipping breakfast would cause obesity may be related to the greater thermogenesis, which was found in those having breakfast regularly (32). Alcohol consumption has probably contributed to the excess energy intake that was associated with weight gain (33). Increased sleep time during childhood has advantageous effects on protecting against a genetic predisposition to obesity, with leptin playing a key role in the process (34). Moreover, excess TV time is frequently associated with prolonged sedentary time and worse dietary behavior, such as taking more fried food when watching TV, which leads to the elevated risk of overweight (35).

There are few studies addressing the associations between oral health and the risk of obesity, especially in children. A cohort study in Japanese adults found that brushing teeth frequently (>3 times/day) may reduce the risk of obesity (OR: 0.49, 95% CI: 0.28–0.85) (36). Our study expands previous findings and showed that unhealthy toothbrushing habit was also a risk factor for overweight/obesity in students. Although the exact mechanism was unclear, obesity has been associated with the altered oral microbiome and periodontal disease (37), and inflammation was proposed as a key feature linking obesity and dental microbial diseases (38). In the animal model, obesity has been reported to interfere with the ability of the immune system to appropriately respond to the infection caused by the periodontal pathogen Porphyromonas gingivalis (39).

Previous studies have identified lifestyle risk factors for overweight/obesity, however, comparisons of attributions may not be applicable due to different study settings, populations and data collection methods. In the present study, we calculated the PAFs after controlling for all the available modifiable lifestyle factors, which allowed us to estimate and compare the attributable effects of these risk factors. The results not only demonstrated the magnitude of the associations for different factors, such as insufficient sleep duration and excess TV time, but also ranked the importance of removing those lifestyle factors for obesity intervention based on estimated rate reduction of overweight/obesity.

A study found that not participating in sports teams had the biggest influence on the rate of obesity in American high school students, followed by watching TV and playing computer games (16). Similarly, this study identified that shortening TV time should be prioritized in obesity intervention among Chinese students. Additionally, promoting sufficient sleeping duration could be the most significant move for obesity control in Chinese students. Furthermore, this study also suggested the ranked risk factors for overweight/obesity varied by sex, age, and region, which warranted population-specific strategies for obesity prevention and control.

Our study had several limitations. First, due to the cross-sectional study design, a causal relationship could not be confirmed. Second, other preventable risk factors or potential confounding factors for obesity, such as mental health status and socioeconomic status, may have not been considered, which could limit the applicability of our findings. Smoking was not included in the analysis due to the low rate in the total participants (~1%). Third, lifestyle factors were collected by self-administered questionnaires, therefore information bias should be considered. Last but not least, only students of Zhejiang province were included in this study, therefore it is unclear to what extent the findings are generalizable to other regions.

## Conclusion

Our study found the prevalence of overweight/obesity in Chinese students could decline moderately if unhealthy lifestyle behaviors, such as insufficient sleep, TV time, and alcohol use, were modified. The attributable risk for obesity of lifestyle behaviors varied in age, sex and residence groups. Findings of this study are important to provide evidence for prioritizing obesity prevention and control initiatives. Population-specific strategies should be considered in future national intervention programs.

## Data Availability Statement

The raw data supporting the conclusions of this article will be made available by the authors. Please contact the corresponding author.

## Ethics Statement

The studies involving human participants were reviewed and approved by Peking University Institutional Review Board. Written informed consent to participate in this study was provided by the participants' legal guardian/next of kin.

## Author Contributions

SZ, BD, and FG made contributions to the conception, design, and acquisition of data. SZ and FG organized the database and drafted the initial manuscript. SZ performed the statistical analysis. BD, JH, KL, XL, and RD wrote sections of the manuscript. All authors contributed to manuscript revision, read, and approved the submitted version.

## Funding

This work was supported by the National Natural Science Foundation of China to Bin Dong (Grants no. 81903344).

## Conflict of Interest

The authors declare that the research was conducted in the absence of any commercial or financial relationships that could be construed as a potential conflict of interest.

## Publisher's Note

All claims expressed in this article are solely those of the authors and do not necessarily represent those of their affiliated organizations, or those of the publisher, the editors and the reviewers. Any product that may be evaluated in this article, or claim that may be made by its manufacturer, is not guaranteed or endorsed by the publisher.
