# Supplementary material for: Lifestyle Risk Factors and the Population Attributable Fractions for Overweight and Obesity in Chinese Students of Zhejiang Province
Source: Front Pediatr. 2021 Sep 22;9:734013. doi: 10.3389/fped.2021.734013 (PMC8493597; doi:10.3389/fped.2021.734013)
Supplement: Supplementary file 1 [file Data_Sheet_1.docx]

**Supplementary Material**

Supplementary Table 1 Population attributable fractions of each risk factor for overweight/obesity (%)

|  | Model 1 | | | Model 2 | | |
| --- | --- | --- | --- | --- | --- | --- |
| variables | PAF | 95% CI | P value | PAF | 95% CI | P value |
| Toothbrushing habit | 0.30 | 0.1,0.6 | 0.006 | 0.3 | 0.0,0.5 | 0.037 |
| Sugar-sweetened beverages | 1.00 | 0.4,1.6 | <0.001 | 0.6 | 0.0,1.2 | 0.039 |
| Fried food | 3.80 | -0.7,8.3 | 0.092 | 1.3 | -3.4,5.8 | 0.589 |
| Breakfast | 1.70 | 1.0,2.4 | <0.001 | 1.2 | 0.5,1.9 | 0.001 |
| Exercise | 0.50 | -0.2,1.3 | 0.195 | 0.3 | -0.4,1.0 | 0.441 |
| Smoke | -0.70 | - | 0.93 | -5.0 | - | 0.543 |
| Alcohol use | 2.00 | 1.0,3.0 | <0.001 | 1.6 | 0.5,2.6 | 0.003 |
| TV time | 3.90 | 2.6,5.2 | <0.001 | 3.5 | 2.2,4.8 | <0.001 |
| Sleep duration | 4.50 | 3.0,6.0 | <0.001 | 4.4 | 2.8,5.9 | <0.001 |

Model 1 was adjusted for age, sex, city, urban/rural area. Model 2 is adjusted by age, sex, city, urban/rural area and all other lifestyle factors.

Supplementary Table 2 Predicting overweight/obesity prevalence if the lifestyle risk factor was eliminated (%)

| variables | Theoretical prevalence | Reduction | 95% CI |
| --- | --- | --- | --- |
| Sleep time | 24.1 | 1.1 | 0.7,1.5 |
| TV time | 24.3 | 0.9 | 0.6,1.2 |
| Alcohol use | 24.8 | 0.4 | 0.1,0.7 |
| Breakfast | 24.9 | 0.3 | 0.1,0.5 |
| Sugar-sweetened beverages | 25.0 | 0.2 | 0.0,0.3 |
| Toothbrushing habit | 25.1 | 0.1 | 0.0,0.1 |

Model is adjusted by age, sex, city, urban/rural area and all the lifestyle factors.
